# Dragon’s Blood-Loaded Mesoporous Silica Nanoparticles for Rapid Hemostasis and Antibacterial Activity

**DOI:** 10.3390/molecules29081888

**Published:** 2024-04-21

**Authors:** Cuiyun Yin, Yihang Li, Jing Yu, Zhaoyou Deng, Shifang Liu, Xuanchao Shi, Deying Tang, Xi Chen, Lixia Zhang

**Affiliations:** 1 Yunnan Branch, Institute of Medicinal Plant, Chinese Academy of Medical Sciences, Jinghong 666100, China; cyyin@implad.ac.cn (C.Y.); jyu@implad.ac.cn (J.Y.); zydeng@implad.ac.cn (Z.D.); sfliu@implad.ac.cn (S.L.); xcshi@implad.ac.cn (X.S.); dytang@implad.ac.cn (D.T.); chenxi@implad.ac.cn (X.C.); 2 Key Laboratory of Sustainable Utilization of Southern Medicine, Jinghong 666100, China

**Keywords:** traditional Chinese medicine, dragon’s blood, mesoporous silica nanoparticles, hemostasis, antibacterial activity

## Abstract

Dragon’s blood (DB) is a traditional Chinese medicine (TCM) with hemostatic effects and antibacterial properties. However, it is still challenging to use for rapid hemostasis because of its insolubility. In this study, different amounts of DB were loaded on mesoporous silica nanoparticles (MSNs) to prepare a series of DB-MSN composites (5DB-MSN, 10DB-MSN, and 20DB-MSN). DB-MSN could quickly release DB and activate the intrinsic blood coagulation cascade simultaneously by DB and MSN. Hemostasis tests demonstrated that DB-MSN showed superior hemostatic effects than either DB or MSNs alone, and 10DB-MSN exhibited the best hemostatic effect. In addition, the antibacterial activities of DB-MSN against *Staphylococcus aureus* (*S. aureus*) and *Escherichia coli* (*E. coli*) improved with the increase in DB. Furthermore, the hemolysis assay and cytocompatibility assay demonstrated that all DB-MSNs exhibited excellent biocompatibility. Based on these results, 10DB-MSN is expected to have potential applications for emergency hemostatic and antibacterial treatment in pre-hospital trauma.

## 1. Introduction

Uncontrolled hemorrhage is a severe global problem that urgently needs to be solved [1,2,3]. Rapid and effective hemostatic agents are crucial in reducing blood loss and mortality [4,5,6]. Traditional Chinese medicine (TCM) is an important part of the prevention and treatment of hemorrhagic diseases [7,8,9]. The hemostatic mechanism of TCM is unique due to its multi-component, multi-target and multi-pathway effects, as shown by modern research [10,11,12]. *Panax notoginseng*, the main component of Yunnan Baiyao, is a famous Chinese hemostatic medicine that can promote platelet aggregation to achieve rapid hemostasis [10]. Liang et. al. reported that carbon dots derived from carbonized *Platycladus orientalis* showed excellent hemostatic effects on exogenous and endogenous hemorrhage through activation of platelets and coagulation pathways [11]. *Bletilla striata* polysaccharide, the main constituent of *Bletilla striata*, has demonstrated excellent blood clotting capability by increasing platelet aggregation rate [12].

Dragon’s blood (DB) is a red resin extracted from the stem of *Dracaena cochinchinensis*. TCM asserts that DB is capable of stopping bleeding, dispersing swelling, and promoting tissue regeneration [13]. DB demonstrates effective hemostatic activity by participating in the endogenous coagulation system. In addition, clinical trials have proved that DB can significantly reduce the probability of rebleeding, exhibit good antibacterial performance, and accelerate wound healing [14,15,16]. However, the use of DB for rapid hemostasis is still greatly challenging due to its hydrophobicity, which results in a low dissolution concentration and a slow dissolution rate.

Mesoporous silica nanoparticles (MSNs) are a potential hemostasis material because of their good adsorption capacity and the high surface concentration of negative net charges and silanol groups [17]. In addition, they are easy to functionalize with many kinds of clotting molecules including metal ions, small organic molecules, and proteins. Functionalized MSNs have been developed for various applications, including increasing antibacterial activity, improving hemostatic performance, and improving biocompatibility [18,19,20]. For example, polyphosphate-functionalized silica nanoparticles were more potent than bare silica nanoparticles at promoting coagulation [18]. Tannic acid-loaded MSNs exhibited better antibacterial activities and could reduce the hemostatic time by 65% [19]. MSNs were coordinated with a glycerol-modified N-alkylated chitosan sponge to develop a rapid hemostatic agent with minimal side effects [20]. These functionalized MSNs had excellent hemostatic effects. However, to the best of our knowledge, no DB-loaded MSNs for hemostasis have been reported.

**Scheme 1:**
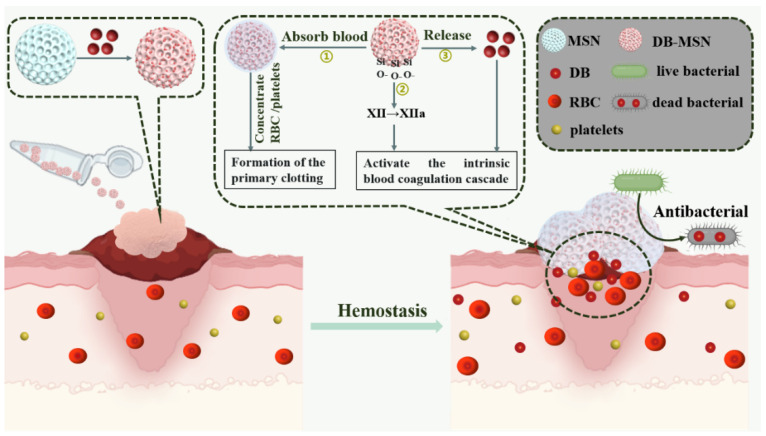
The schematic illustration of the DB-MSN for rapid hemostasis and antibacterial activity.

Here, we combined DB and MSNs to develop a rapid hemostatic and antibacterial agent with the advantages of both materials. As shown in Scheme 1, the hemostatic mechanism of DB-MSN was attributed to the fact that (1) it provided excellent blood absorption because of MSNs with their large specific surface and porous structure, (2) it could bind clotting factor XII via the negatively charged silanol surface of the MSNs, and (3) it could quickly release DB and then activate the intrinsic blood coagulation cascade. In order to examine the possibility of DB-MSN as a hemostasis and antibacterial agent, the morphology, physicochemical properties, loading capacity, and DB-release behavior of DB-MSN were studied. Then, the hemostatic ability of DB-MSN was researched in detail by clotting time, APTT, PT, a rat tail amputation model, and a rat liver injury model. In addition, bacteriostatic experiments, hemolysis, and live/dead staining experiments were performed to evaluate the antibacterial activity and biocompatibility of DB-MSN.

## 2. Results and Discussion

### 2.1. The Characterization of MSN, 5DB-MSN, 10DB-MSN, and 20DB-MSN

**Figure 1:**
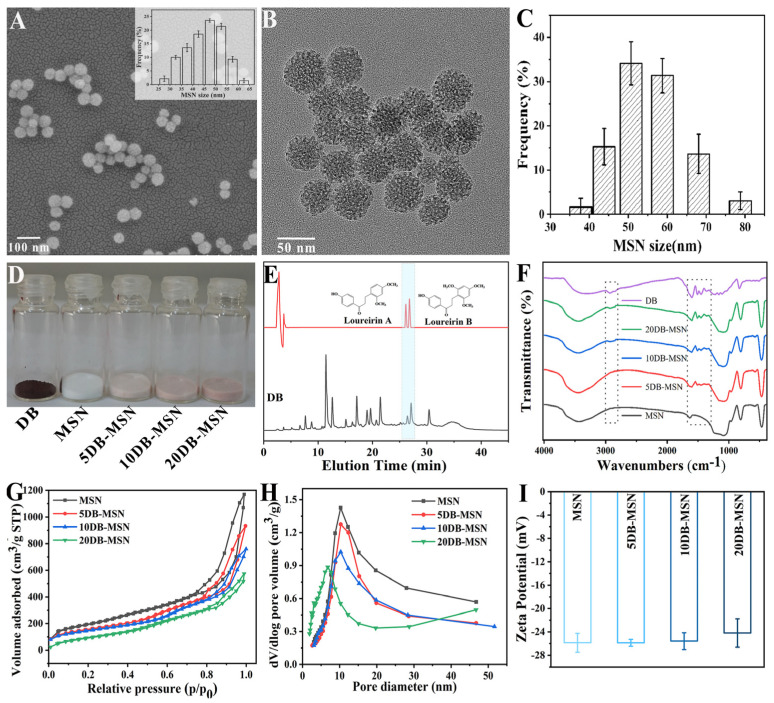
The characterization of MSNs and DB-MSN. (**A**) SEM image of MSNs, the inset shows the size distribution of MSNs. (**B**) TEM image of MSNs. (**C**) The size distribution of MSNs by DLS in ethanol. (**D**) Photograph of DB, MSNs, and DB-MSN. (**E**) Liquid chromatogram of DB, Loureirin A, and Loureirin B. (**F**) FT-IR spectrum of MSNs and DB-MSN. (**G**) N_2_ adsorption/desorption isotherms of MSNs and DB-MSN. (**H**) The corresponding pore-size distribution of MSNs and DB-MSN. (**I**) Zeta potential of MSNs and DB-MSN.

The surface morphology and particle size of MSNs were analyzed by SEM and TEM. The MSNs were monodisperse spheres with a mean particle size of 45 nm from the SEM micrograph in Figure 1A. The TEM images revealed that the pores with mean sizes of 4 nm were both on the particle surface and within the particles, as shown in Figure 1B. The DLS results showed the MSNs possessed good dispersion in ethanol, and the MSN mean particle size was 55 nm (Figure 1C).

To prepare the DB-loaded MSNs (DB-MSN) with different weight ratios, DB and MSNs were mixed in ethanol with weight ratios of 5%, 10%, and 20%, and then called 5DB-MSN, 10DB-MSN, and 20DB-MSN, respectively. The prepared MSN, 5DB-MSN, 10DB-MSN, and 20DB-MSN are shown in Figure 1D. Compared to MSN, the color of DB-MSN gradually turned red with the increase in DB because of the dark red DB. The apparent peaks of Loureirin A and Loureirin B, which were two major active compounds of DB, could be observed in chromatograms of DB (Figure 1E). From the standard curves of Loureirin A and Loureirin B (Figures S1 and S2), it can be calculated that Loureirin A and Loureirin B constituted 0.48% and 0.85% of DB, respectively.

The incorporation of DB and MSNs was qualitatively confirmed by FT-IR spectra. As shown in Figure 1F, MSNs displayed a typical FT-IR spectrum of mesoporous silica [19]. The peaks at 3430 cm^−1^ were due to the stretching vibrational frequency of the silanol groups. Two bands of CH_2_ stretching were observed at 2933 cm^−1^ and 2871 cm^−1^. The vibrations associated with the hydrogen bond of n(–OH) in physisorbed water molecules and n(–Si–OH) in free silanol were observed at 1634 cm^−1^ and 954 cm^−1^, respectively. The spectral peaks at 1084 cm^−1^, 800 cm^−1^, and 465 cm^−1^ were assigned to Si–O–Si asymmetric stretching vibration, symmetric stretching vibration, and bending vibration. In the FT-IR spectrum of DB, the characteristic peaks at 1600 cm^−1^, 1510 cm^−1^, and 1452 cm^−1^ were consistent with aromatic C–C stretches, which originated from corresponding to the benzene rings of the main components of DB, flavonoids [21]. For DB-MSN, the characteristic peaks of DB could be observed at 1600 cm^−1^, 1510 cm^−1^, and 1452 cm^−1^, which suggested the successful deposition of DB on the MSN.

**Figure 2:**
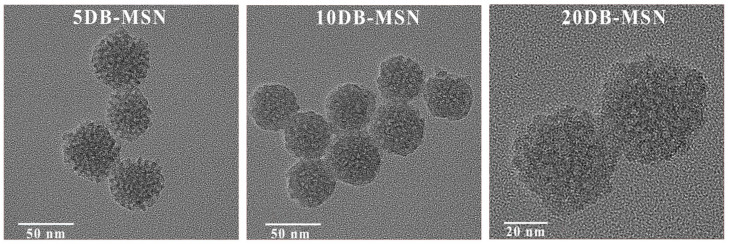
TEM image of 5DB-MSN, 10DB-MSN, and 20DB-MSN.

The impregnation of MSNs by DB was also well supported by the nitrogen adsorption study. The nitrogen adsorption/desorption isotherms and pore-size distributions of the samples are presented in Figure 1G and Figure 1H, respectively. The MSNs and DB-MSN all revealed typical type IV isotherms, which are one of the main characteristics of mesoporous materials. The BET surface areas (S_BET_) of the MSNs were 702 m^2^ g^−1^, the total pore volume (V_t_) was 2.27 cm^3^ g^−1^, and the BJH pore diameter (D_BJH_) was 12.96 nm. Meanwhile, after DB absorption, the S_BET_, V_t_, and D_BJH_ of 5DB-MSN, 10DB-MSN, and 20DB-MSN decreased to 567 m^2^ g^−1^, 2.06 cm^3^ g^−1^, and 11.43 nm; 427 m^2^ g^−1^,1.70 cm^3^ g^−1^, and 9.47 nm; as well as 248 m^2^ g^−1^, 0.92 cm^3^ g^−1^, and 5.43 nm, respectively (Table 1). It was clear that S_BET_, V_t_, and D_BJH_ were reduced with the increase in DB. The reason for this phenomenon may be that the filled degree of the pores increased with the increase in DB because the DB was loaded into the pores of the DB-MSN. The TEM results (Figure 2) visually showed that the pores of MSNs were gradually filled up as DB increased.

The zeta potentials of MSNs were determined before and after loading DB, as shown in Figure 1I. The zeta potential of the MSNs before loading DB was −25.87 mV, while when loading DB, the zeta potentials of 5DB-MSN, 10DB-MSN, and 20DB-MSN were −25.88 mV, −25.58 mV, and −24.19 mV, respectively. No significant change could be observed before or after DB loading. The results indicated that the external surface of the MSNs did not undergo significant modification after DB loading, and DB was mostly located inside the pores of MSN.

### 2.2. The Loading Capability of DB-MSN and DB Release from DB-MSN In Vitro

**Figure 3:**
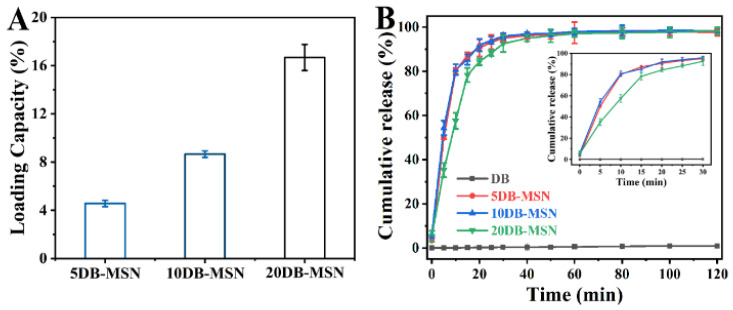
(**A**) Loading capacity of DB-MSN. (**B**) DB-release behaviors of DB-MSN in PBS solution.

The loading capability of DB on the MSNs and the release of DB from DB-MSN were quantified by the UV-vis absorption value of DB at 284 nm (Figure S3). The loading capabilities of 5DB-MSN, 10DB-MSN, and 20DB-MSN using a mass ratio were calculated as 4.57%, 8.76%, and 16.68%, respectively (Figure 3A). It was clear that the loading capability increased with the higher mass ratio of DB to MSNs when the amount of the MSNs was fixed. This is because the uptake of DB mainly depends on the pore size, surface area, and mesoporous structure of MSN, as indicated in previous reports [22,23].

The release profiles of DB from pure DB, 5DB-MSN, 10DB-MSN, and 20 DB-MSN in a PBS solution are presented in Figure 3B. There were major differences between the DB release curves of DB-MSN and pure DB. The release rate of the pure DB was quite low, and the cumulative release of DB at 120 min was no more than 2%. However, the release reached equilibrium at sampling times of 30 min. The cumulative releases of 5DB-MSN, 10DB-MSN, and 20DB-MSN were 97.60%, 98.37%, and 98.32%, respectively. The dissolution improvement of DB may be attributed to the mesopores of MSNs, which (1) changed the crystalline state of DB to a noncrystalline state and (2) increased the contact area between the DB and the release medium [21]. The release profiles of all DB-MSN samples were of the same type, with a high release rate during the initial 15 min, followed by a slow, sustained release of DB. The release kinetics of DB-MSN being the same may be because (1) DB in the external pores of the MSNs could be released quickly into the release medium but DB in the pores inside the particles was released along with their slow dissolution [24]; (2) the narrowing concentration difference during release further contributes to this release behavior. It is worth noting that the release rate of 20DB-MSN was lower than that of 5DB-MSN and 10DB-MSN at the initial stage; the cumulative release of 5DB-MSN, 10DB-MSN, and 20DB-MSN at sampling times of 15 min reached 87.23%, 85.36%, and 78.32%, respectively. This was probably because the distribution of DB on the pores of 20DB-MSN was greater than that of 5DB-MSN and 10DB-MSN, resulting in a smaller contact surface.

### 2.3. Hemostatic Efficiency Evaluation In Vitro

**Figure 4:**
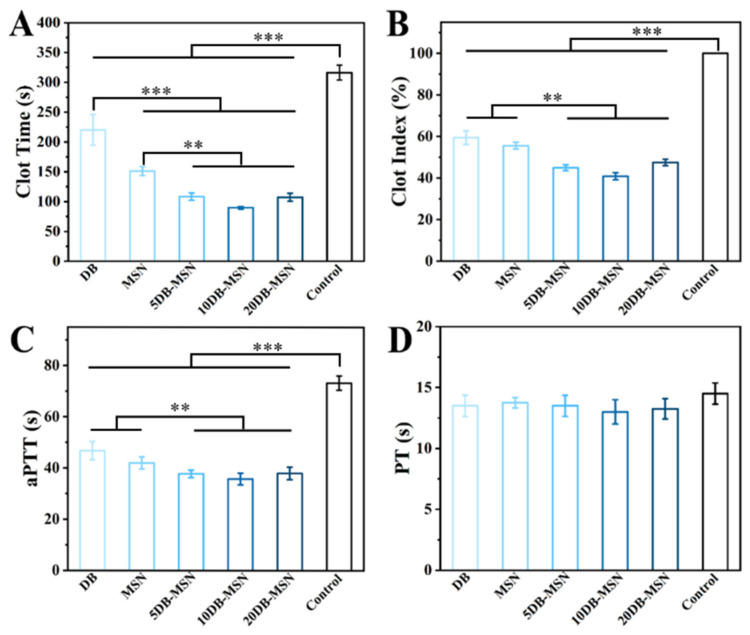
Analysis of hemostatic efficiency in vitro. (**A**) CT results for the control, DB, MSNs, and DB-MSN. (**B**) CI results for the control, DB, MSNs, and DB-MSN. (**C**) APPT results for the control, DB, MSNs, and DB-MSN. (**D**) PT results for the control, DB, MSNs, and DB-MSN. These data are represented as the mean ± SD (n = 3). (**, *p* < 0.01; ***, *p* < 0.001).

Clotting time (CT) is an intuitive method to evaluate the hemostatic properties of DB-MSN. As shown in Figure 4A, the CTs of the DB group (220.33 ± 25.95 s) and the MSN group (151.33 ± 7.41 s) were significantly shorter than the control group (316.33 ± 7.41 s), indicating that both DB and MSNs can effectively promote blood coagulation [14,25]. The CT of 5DB-MSN, 10DB-MSN, and 20DB-MSN were approximately 108.33 ± 6.23 s, 89.67 ± 2.05s, and 107.33 ± 6.55s, respectively, faster than that of MSNs and DB. The 10DB-MSN showed a shorter CT than the 20DB-MSN; this phenomenon may be related to the higher release rate of 10DB-MSN than 20DB-MSN at the initial stage. The clotting index (CI) with scientific rationality was also used to evaluate the hemostatic efficiency (Figure 4B). Compared to the control group (set to 100%), the CI of DB and MSNs were 59.39 ± 3.29% and 55.56 ± 1.57%, respectively. DB-MSN showed a smaller CI than DB and MSN, and the CIs of 5DB-MSN, 10DB-MSN, and 20DB-MSN were 44.92 ± 1.44%, 40.81 ± 1.68%, and 47.47 ± 1.57%, respectively. The CI results were consistent with the CT results. Overall, the DB-MSN performed better hemostatic efficiency than pure DB or MSN, and the performance of the 10DB-MSN was optimal.

The activated partial thromboplastin time (aPTT) and prothrombin time (PT) are correlated with the intrinsic coagulation pathway and the extrinsic pathway, respectively. As shown in Figure 4C, the aPTT values of DB (46.75 ± 3.56 s), MSNs (42 ± 2.34 s), 5DB-MSN (37.75 ± 1.48 s), 10DB-MSN (35.75 ± 2.28 s), and 20DB-MSN (38 ± 2.45 s) were significantly reduced compared with the control group (73.25 ± 2.77 s). Compared with MSNs and DB, the aPTT value of DB-MSN was significantly reduced, but there was no significant difference in the aPTT value between 5DB-MSN, 10DB-MSN, and 20DB-MSN. In addition, the PT of all the groups remained unchanged (Figure 4D), indicating that DB-MSN had no influence on the extrinsic coagulation pathway.

From the above results, the hemostatic efficiency of DB-MSN was better than that of MSNs and DB. The improved hemostatic efficiency of DB-MSN was attributed to DB-MSN could integrate the hemostatic advantages of both materials. DB-MSN not only provided excellent blood absorption and a negatively charged silanol surface because of the MSNs, but also quickly released DB, which can activate the blood coagulation cascade. The results of different DB-MSNs indicated the content of DB and the kinetics of DB release were the factors affecting DB-MSN hemostatic efficiency. DB-MSN with higher DB content means more DB is available for hemostasis, but higher DB content of DB-MSN with a slower release rate at the initial stage (Figure 3B) leads to less DB playing a role in actual hemostasis.

### 2.4. Hemostatic Efficiency Evaluation in Animal Models

**Figure 5:**
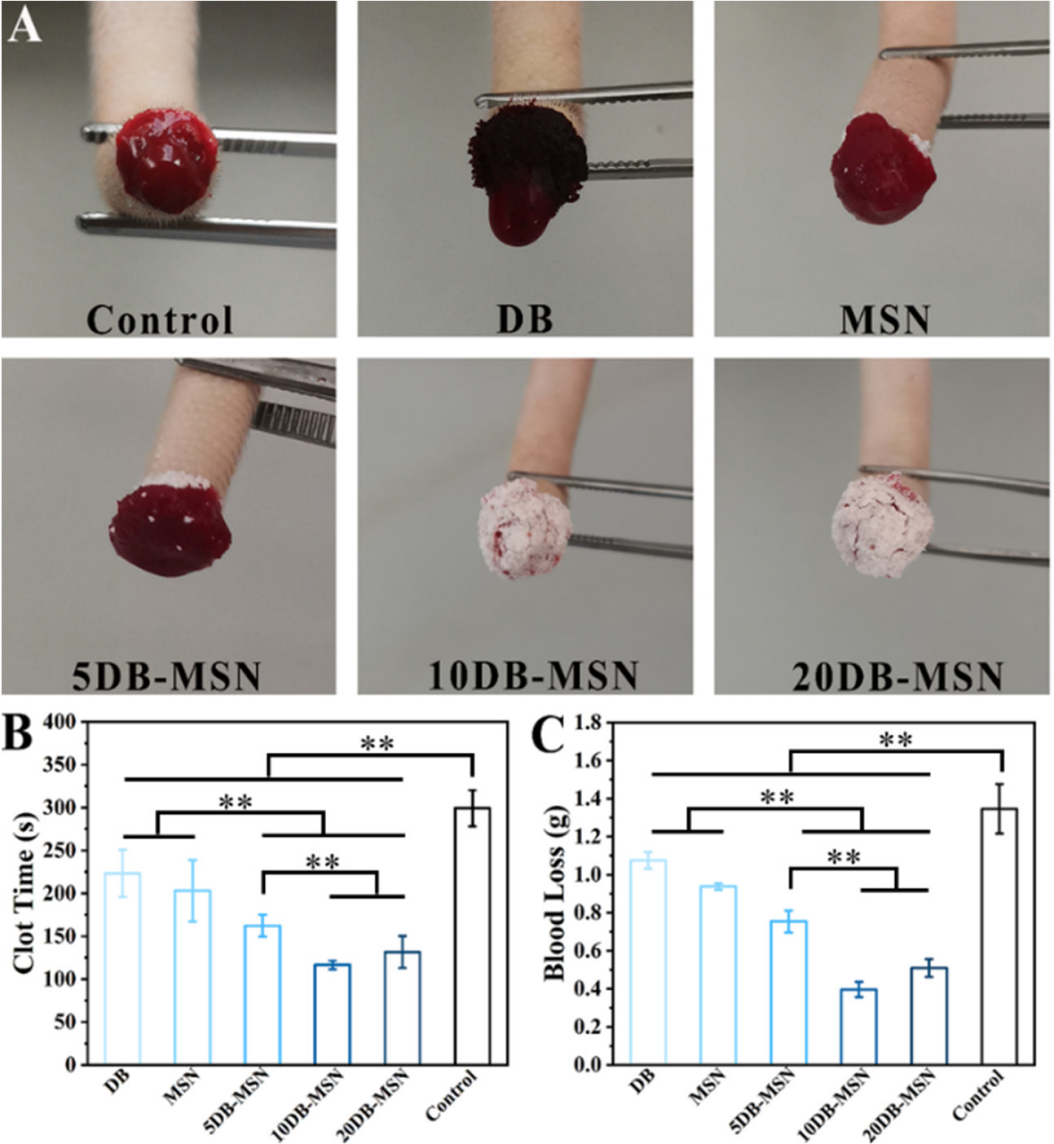
Analysis of hemostatic efficiency in the rat tail amputation models. (**A**) Photograph of the rat tail amputation models when bleeding stopped. (**B**) Hemostasis time in the rat tail amputation model. (**C**) Blood loss in the rat tail amputation model. These data are represented as the mean ± SD (n = 3). (**, *p* < 0.01).

The rat tail amputation models were adopted to evaluate the in vivo hemostatic efficiency of DB-MSN. As shown in Figure 5A, after hemostasis, a large blood clot was observed in the negative control group, while no apparent blood clot was formed in the 10DB-MSN and 20DB-MSN groups. For the DB, MSN, and 5DB-MSN groups, blood clots were formed on the surface of the materials. In addition, hemostasis time and blood loss in the different groups were recorded (Figure 5B,C). The hemostasis time for the negative control group was about 237.67 ± 6.03 s. The hemostasis time was reduced to 142.67 ± 13.20 s and 154.67 ± 6.81 s when MSNs and DB were applied, respectively. Specifically, the 5DB-MSN, 10DB-MSN, and 20DB-MSN showed a significant reduction of about 121.00 ± 8.00 s, 81.00 ± 5.57 s, and 86.67 ± 1.53 s, respectively. The blood loss of 10DB-MSN and 20DB-MSN was the least, about 0.396 ± 0.040 g and 0.510 ± 0.047 g, respectively. The blood loss of 5DB-MSN (0.754 ± 0.058 g), MSNs (0.938 ± 0.017 g) and DB (1.075 ± 0.043 g) was larger than that of 10DB-MSN and 20DB-MSN. The 10DB-MSN and 20DB-MSN with minimal bleeding time and blood loss could be attributed to 10DB-MSN and 20DB-MSN could adhere to the bleeding surface and stop bleeding immediately. These above results suggested that the prepared DB-MSN greatly improved hemostatic performance compared to the single materials, and 10DB-MSN showed the best hemostatic efficiency.

**Figure 6:**
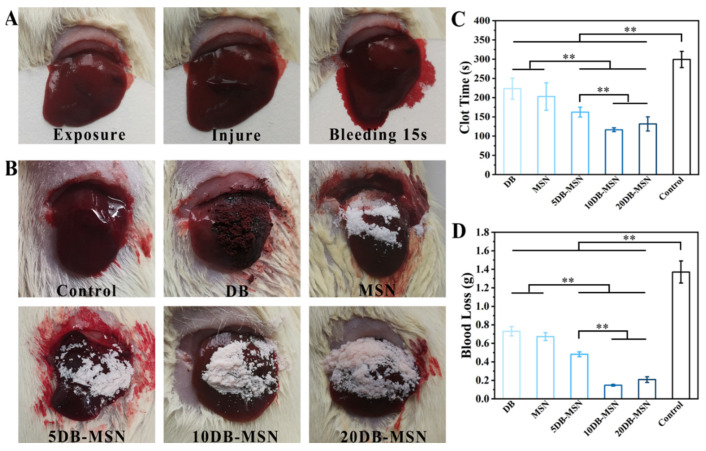
Analysis of hemostatic efficiency in the rat liver injury models. (**A**) The process of constructing the rat liver injury models. (**B**) Photograph of the rat liver injury models when bleeding stopped. (**C**) Hemostasis time in the rat liver injury model. (**D**) Blood loss in the rat liver injury model. These data are represented as the mean ± SD (n = 3). (**, *p* < 0.01).

To further investigate the rapid and effective hemostatic ability of DB-MSN, the rat liver injury models were constructed as shown in Figure 6A. The exposed rat livers were cut into a wound of 1 cm in length and 0.3 cm in depth using a scalpel. After 15 s of free bleeding, 40 mg samples were immediately coated on the wound site. These results of complete hemostasis are shown in Figure 6B. The hemostatic times of the MSNs and DB were 203.00 ± 35.79 s and 223.33± 27.54 s, respectively, which were obvious shorter than the control group without hemostatic treatment (299.33 ± 21.00 s). The hemostatic times of 5DB-MSN, 10DB-MSN, and 20DB-MSN were significantly shortened to 162.33 ± 12.85 s, 116.67 ± 4.93 s, and 131.67 ± 18.50 s, respectively (Figure 6C). The control group showed the biggest blood loss of up to 1.37 ± 0.12 g. DB, MSNs, and 5DB-MSN significantly decreased the blood loss to 0.73 ± 0.05 g, 0.67 ± 0.04 g, and 0.482 ± 0.02 g, respectively. The blood loss had no obvious difference between the 10 DB-MSN group (0.14 ± 0.01 g) and the 20DB-MSN group (0.21 ± 0.03 g) (Figure 6D). These results further verified that the prepared DB-MSN greatly improved hemostatic performance, and 10DB-MSN showed the best hemostatic efficiency.

### 2.5. Antibacterial Performance

**Figure 7:**
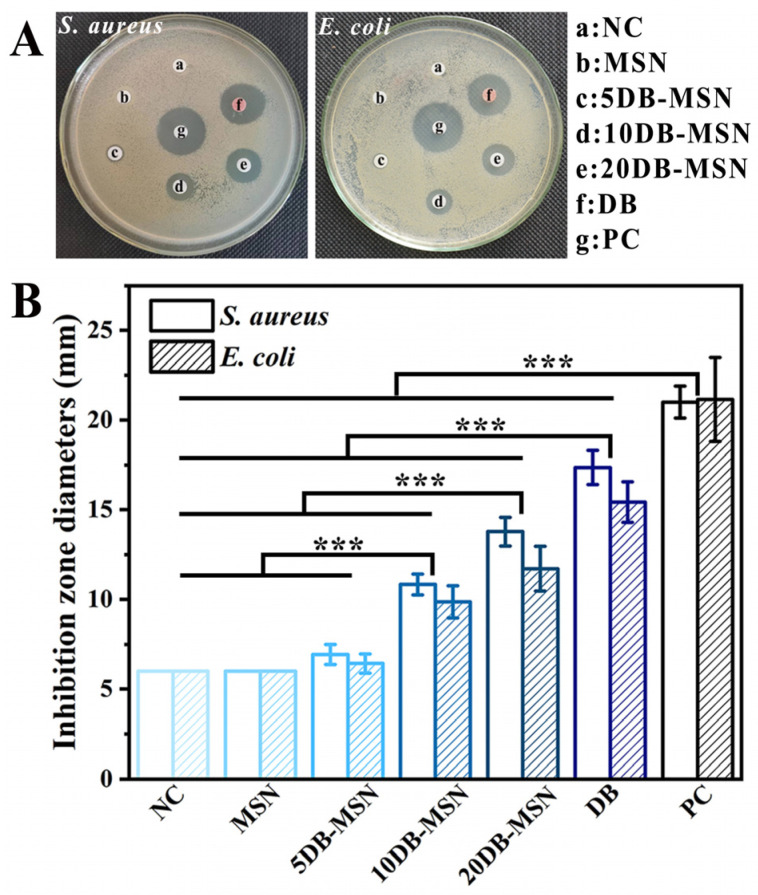
Antibacterial performance analysis. (**A**) The photographs of inhibition zones after bacterial and various samples co-cultured for 24 h. (**B**) The diameters of inhibition zones for the various samples. These data are represented as the mean ± SD (n = 7). (***, *p* < 0.001).

The antibacterial activity of hemostatic material is an essential factor in wound healing after hemostasis. To evaluate the antibacterial performance of DB-MSN, the inhibition zones of different samples for *S. aureus* (Gram-positive bacteria) and *E. coli* (Gram-negative bacteria) were tested. As shown in Figure 7A,B, inhibition zones around blank paper (No. a sample) and MSN-coated paper (No. b sample) were not observed, which indicated blank paper and MSNs were devoid of antimicrobial activity. A narrow inhibition zone over the 5DB-MSN-coated paper (No. c sample) against *S. aureus* and *E. coli* was 6.9 ± 0.6 mm and 6.4 ± 0.5 mm, respectively. Compared to that of the 5DB-MSN-coated paper, the inhibition zone of the 10DB-MSN-coated paper (No. d sample) and 20DB-MSN-coated paper (No. e sample) against *S. aureus* and *E. coli* significantly increased 10.8 mm and 9.86 mm, 13.8 mm and 11.7 mm, respectively. It can be observed that clear inhibition zones were formed by increasing the DB concentration of the DB-MSN. DB-coated paper (No. f sample) demonstrated good antibacterial levels against *S. aureus* and *E. coli* with inhibition zones of 21.0 ± 0.9 mm and 17.4 ± 1.0 mm, respectively. The positive control groups, streptomycin-coated paper (No. g sample), demonstrated good antibacterial levels against *S. aureus* and *E. coli* (inhibition zones of 21.0 ± 0.9 mm and 17.4 ± 1.0 mm, respectively). The minimal inhibitory concentration (MIC) results of DB-MSN (Table S1) showed a similar trend as the inhibition zone results; the inhibitory activity of DB-MSN increased with the amount of DB. These results demonstrated that DB-MSN displayed good antibacterial activity, and their antibacterial activity was related to the amount of DB. The antibacterial activity of the DB-MSN could be attributed to the active ingredient DB released from the DB-MSN, which can reduce the biofilm formation of bacteria [26].

### 2.6. Biocompatibility Analysis

**Figure 8:**
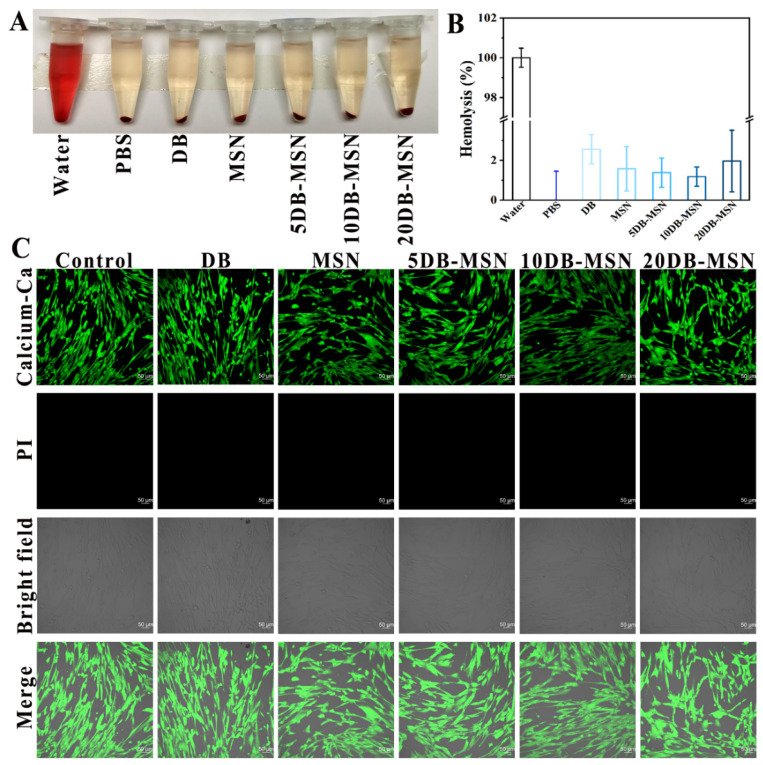
Biocompatibility analysis. (**A**) Hemolysis photograph of the control, DB, MSNs, and DB-MSN. (**B**) Hemolysis ratio of the control, DB, MSNs, and DB-MSN. Values correspond to the mean ± SD (n = 3). (**C**) Live/dead staining of cells incubated with the control, DB, MSNs, and DB-MSN. The sample concentrations were all 0.64 mg/mL. Scale 50 μm.

Blood compatibility is quite important for hemostatic materials, which are directly in contact with the wound and mixed with blood [20]. A hemolysis assay was performed as an easy and trustworthy method to evaluate the blood compatibility of hemostats [19]. Figure 8A shows the results of the hemolysis assay. The distilled water group (positive control group) was red with hemolysis, while the PBS (negative control group), DB, MSN, 5DB-MSN, 10DB-MSN, and 20DB-MSN were clear and transparent without hemolysis. Figure 8B shows the hemolysis ratios of DB (2.56 ± 0.74%), MSNs (1.58 ± 1.12%), 5DB-MSN (1.38 ± 0.74%), 10DB-MSN (1.18 ± 0.48%), and 20DB-MSN (1.97 ± 1.55%), respectively. The hemolysis ratios of DB-MSN were less than 5%, which reached the safety level of hemostatic materials, which indicated that 5DB-MSN, 10DB-MSN, and 20DB-MSN had excellent blood compatibility.

Live/dead staining of HFL-1 fibroblasts was employed to visually validate the cytocompatibility of samples. As shown in Figure 8C, all sample groups exhibited bright green fluorescence similar to that of the control group. The cell morphology and cell death of all groups, including the sample groups and the control group, were similar. The result confirmed that DB-MSN possessed good cytocompatibility.

In summary, the results above proved that the DB-MSN could integrate the advantages of DB and MSN and provide efficient hemorrhage control, excellent antibacterial properties, and good biocompatibility. Only rat tail amputation and rat liver injury were used to evaluate hemostasis in vivo for the restriction of experimental conditions, which may lead to potential limitations in regard to human hemostasis because of disparities in rat and human physiology. In addition, based on the previous reports and the current study findings here, the surface characteristics (pore size, surface area, and mesoporous structure) of MSNs not only affect the uptake and release behaviors of DB but are also closely related to its hemostatic effect. Therefore, the correlation between the surface characteristics of the MSNs and the hemostatic performance of DB-MSN is a potential avenue for further research.

## 3. Materials and Methods

### 3.1. Materials

Hexadecy ltrimethyl ammonium bromide (CTAB), tetraethyl orthosilicate (TEOS), methyl methacrylate (MMA), L-lysine, octane, 3-aminopropyltriethoxysilane (APTES), and 2, 2′-Azobis (2-methylpropionamide) dihydrochloride (AIBA) were purchased from Adamas (Shanghai, China). Dragon’s blood was obtained from Jianlong Pharmaceutical Co. Ltd. (Jinghong, China). Deionized water was used throughout this work, and all other chemicals were of analytical grade. The strains of *S. aureus* (CICC 21600) and *E. coli* (CICC 10899) were purchased from the China Center of Industrial Culture Collection (Beijing, China).

### 3.2. Preparation of MSNs and DB-MSN

**Preparation of MSNs** MSNs were synthesized using an organic template method [27]. First, 300 mg of CTAB was dissolved in 96 mL of deionized water at 70 °C with constant stirring and nitrogen for around 2 h until a clear solution was obtained. Then, 7 mL of octane was added to the clear solution, followed by stirring for 30 min. After that, 66 mg of L-lysine, 3000 mg of TEOS, 60 mg of MMA, and 0.81 mg of AIBA were slowly added to the solution. The solution was stirred at 600 rpm for 4 h under nitrogen at 70 °C, resulting in a one-phase, milky white solution. The system was then cooled to room temperature and left for 12 h. The resulting solution was purified by centrifugation at 20,000 rpm, with ethanol used to wash the centrifuged particles. The organic template was completely removed by heat treatment at 600 °C for 5 h in the air, resulting in the formation of white and dry MSN powder.

**Preparation of DB-MSN** DB-loaded MSNs were prepared by taking the following steps: Different amounts of dragon’s blood were dissolved in 10 mL of alcohol to prepare different concentrations of DB solutions. Then, 100 mg of as-synthesized MSN powder was added to a certain concentration of DB solution and mixed by stirring for 3 h. After stirring, the samples were centrifuged and dried at 60 °C for 5 h. Eventually, 5DB-MSN, 10DB-MSN, and 20DB-MSN were obtained, where 5, 10, and 20 represent the theoretical percentages of DB loaded on the MSNs.

### 3.3. Characterization

The surface morphology of the samples was analyzed by scanning electron microscopy (SEM, Hitachi SU8100, Tokyo, Japan, operating at 3 kV) and transmission electron microscopy (TEM, JEOL JEM-F200, Akishima, Japan, operating at 200 kV). For SEM measurements, the samples were deposited on the carbon tape, and then gold sputtering was carried out. For TEM measurements, the samples were ultrasonically dispersed in ethanol for 5 min and dripped onto a copper mesh covered with carbon.

Dynamic light scattering (DLS) was employed to measure the size of particles and their surface charge using a Zetasizer Nano ZS90 instrument (Malvern, Worcestershire, UK). The zeta potential was determined by analyzing diluted suspensions in H_2_O with a pH value of 7.0.

To determine N_2_ adsorption-desorption isotherms and pore size distributions, an ASAP 2460 instrument (Micromeritics, Atlanta, GA, USA) was utilized. Prior to analysis, the MSN and DB-MSN samples were subjected to degassing at temperatures of 350 °C and 40 °C under vacuum for durations of 12 h, respectively. The specific surface area was calculated using the Brunauer-Emmett-Teller (BET) method, while pore parameters such as volume and diameter were obtained based on the Barrett-Joyner-Halenda (BJH) model by examining the adsorption branch.

Fourier transform infrared (FT-IR) spectra were recorded at room temperature using a Tensor 27 spectrometer (Bruker, Billerica, Germany). For measurements, KBr was mixed with the samples, followed by lamination. The FT-IR spectra covered a range of 400–4000 cm^−1^ with a resolution set at 2 cm^−1^.

UV-vis spectra were used to determine the concentration of DB using a UV-vis spectrophotometer (Hitachi 5300, Tokyo, Japan); the peak at approximately 285 nm represented the DB.

### 3.4. Loading Efficiency and DB-Release Behavior Study

**Loading efficiency** The loading efficiency of DB-MSN was estimated by measuring the amount of free DB in solution after the preparation of DB-MSN. To quantify free DB in solution, the centrifuged solution in “Section 3.2” was collected and then measured for absorbance at 285 nm (the characteristic absorbance of DB). The concentrations were calculated according to the standard curve of DB. The loading efficiency (*LE*) of DB in the MSNs was calculated using the following formula.

$$LE\left(\%\right)=\frac{m_{Theoretical\;drug\;weight}-V_{Reaction\;volume}\times c_{Supernatant\;drug\;concentration}}{m_{The\;total\;weight\;of\;theoretical\;drug\;and\;carrier}}\times 100\%$$

**DB-release behaviors study** The DB-release experiment was carried out using the USP II paddle method. First, 50 mg DB-MSN or DB was added to 25 mL of the phosphate buffer solution (PBS, 0.1 mol L^−1^, pH = 7.4) with 0.1% sodium dodecyl sulfate (SDS), respectively. This mixture was shaken at 100 rpm at 37 °C. Next, 3 mL samples were taken from the released medium at specific time intervals, and an equal volume of fresh medium was added to ensure a consistent dissolution volume. The concentration of DB in the samples was subsequently measured using UV–visible spectroscopy at a wavelength of 285 nm. The cumulative DB release was calculated using the following equation:

$$E_{r}=\frac{V_{e}\sum_{1}^{n-1}C_{i}+V_{0}C_{n}}{m_{DB}}\times 100\%$$

where the cumulative release (%) of DB is denoted as *E_r_*; the volume taken at predetermined time intervals is represented as *V_e_* (*V_e_* = 3.0 mL); the concentration of DB in the released fluid at time i is indicated as *C_i_*; the volume of the released solution is *V*_0_ (*V*_0_ = 25 mL); the number of samples is n; the total amount of DB encapsulated within the MSNs is denoted as *m_DB_*. This determination process underwent three repetitions.

### 3.5. Hemostatic Efficiency Evaluation In Vitro

**Preparation of blood samples and related components** The citrated whole blood of a rabbit, with a blood-to-coagulant ratio of 9:1, was purchased from Shanghai Xinfan Biology Science and Technology Co. Ltd. (Shanghai, China). The centrifugation process involved spinning the whole blood at 3000 rpm for 15 min. The supernatant was platelet-poor plasma (PPP) and the precipitate was red blood cells (RBCs). The RBCs were thoroughly washed using PBS until the supernatant exhibited a bright yellow color, following which they were dispersed with PBS at a concentration of 5% (*v*/*v*) to obtain a pure dispersion of red cells.

**CT** Initially, a 10 mg sample was introduced into a 2 mL plastic tube and incubated at a temperature of 37 °C for a duration of 5 min. No additional substances were added to the control group. Subsequently, the sample was mixed with 1 mL of whole blood and incubated at the same temperature for 3 min. Following this, the tube received an addition of 500 µL of CaCl_2_ solution with a concentration of 0.025 mol L^−1^. The tube was then taken out of the water bath and gently inverted every 10 s until a complete cessation in the flow of blood aggregates occurred. Each group underwent measurement at least three times.

**CI** Initially, a 10 mg sample was placed into a 50 mL plastic tube and subjected to incubation at 37 °C for 5 min. No supplementary substances were included in the control group during this process step. Next, both whole blood (100 µL) and CaCl_2_ solution (20 µL 0.025 mol L^−1^) were mixed with the sample and incubated at 37 °C for 3 min. Following these steps, 25 mL of deionized water was carefully poured into the tube, followed by centrifugation performed at 3000 rpm for 10 min. The absorbance of the supernatants after centrifugation was measured at 540 nm. Each group underwent measurement at least three times. The CI was calculated using the following formula:

$$CI\left(\%\right)=\frac{A_{Sample}}{A_{Control}}\times 100\%$$

**APTT** A 2 mg sample was placed in a 2 mL plastic tube and incubated at 37 °C for 5 min. No additional substances were introduced to the control group. Subsequently, the tube was supplemented with 100 µL of PPP and 100 µL of the aPPT reagent, followed by another incubation period at 37 °C for 5 min. Next, the test tube received an addition of 100 µL of CaCl_2_ solution (0.025 mol L^−1^). The tube was then gently inverted every 10 s until complete cessation of blood aggregation occurred. Each group underwent measurement at least three times.

**PT** The separate tubes containing PPP (100 μL), PT reagent (200 μL), and the sample (2 mg) were incubated at 37 °C for 3 min. Following this step, both the PT reagent and the sample were added to the PPP-containing tube. Negative control tests were conducted without any samples present. Similar to previous steps, inversion occurred every 10 s until blood aggregation ceased completely after removal from the water bath environment.

### 3.6. Hemostatic Efficiency Evaluation In Vivo

This experiment was conducted in strict accordance with the guidelines provided by the National Institutes of Health (NIH) for the ethical care and use of laboratory animals (NIH publication No. 85-23 Rev. 1985). Male Sprague-Dawley (SD) rats weighing between 180 and 220 g were procured from Beijing Huafukang Biotechnology Co., Ltd., Beijing, China. Before the experiments, the animals were fed for 7 d in the experimental environment, and the samples were sterilized via UV irradiation for 2 h. SD rats were anesthetized by an intraperitoneal injection of 40 mg kg^−1^ sodium pentobarbital in the course of the experiments.

**Rat tail amputation model** Briefly, the tails of anesthetized SD rats were disinfected with 75% ethanol. Petri dishes with filter papers were placed under the rat tail, and fifty percent of the length of the tail was cut off using surgical scissors. After 15 s of free bleeding, a 50 mg sample was applied to the bleeding site. The rats without any treatment were set as negative controls. Three batches were repeated. Meanwhile, the bleeding time and blood loss were recorded during the hemostatic process.

**Rat liver injury model** Briefly, the belly hair of anesthetized SD rats was removed, and then the abdominal cavity of detersile SD rats was opened to expose the liver. Filter papers were placed under the liver. A wound of 1 cm in length and 0.3 cm in depth was formed in the middle of the liver using a scalpel causing bleeding. After 15 s of free bleeding, a 50 mg sample was applied to the bleeding site. The rats without any treatment were set as negative controls. Three batches were repeated. Meanwhile, the bleeding time and blood loss were recorded during the hemostatic process.

### 3.7. Antibacterial Assay In Vitro

**Agar disc diffusion method** The inhibition zone diameters for *S. aureus* (Gram-positive bacteria) and *E. coli* (Gram-negative bacteria) were determined by using the agar disc diffusion method [28]. To prepare nutrient agar, a mixture of sodium chloride, plant peptone, agar, and peptone was dissolved in distilled water at a pH of 7.2. Subsequently, 100 μL of a 1 × 10^8^ CFU/mL bacterial suspension was added to 10 mL lysogeny broth agar medium and incubated at 37 °C for 18 h. Filter papers with a diameter of approximately 6 mm were coated by adding 10 μL of a 10 mg/mL sample solution and sterilized using overnight ultraviolet exposure. Equal volumes of 10 μg/mL streptomycin and deionized water were set as positive controls and negative controls, respectively. The bacterial solution was evenly spread on coagulated nutrient agar plates, followed by the placement of the coated testing sample papers on their surfaces. The plates were then incubated at 37 °C for 24 h. Following this process, the diameter of the inhibition zone was measured.

**Determination of MIC** The determination of the MIC of the samples against *S. aureus* was carried out by the broth microdilution method [29]. DB and DB-MSN were dissolved in ethanol and diluted with culture broth to a concentration of 20 mg/mL. Concentrations ranging from 20 to 0.3125 mg/mL in culture medium were obtained through serial dilutions at a ratio of 1:2. 100 μL of each dilution was dispensed into 96-well plates. Sterility control consisted only of culture medium, while growth control contained culture medium plus ethanol. In each well, a microbial suspension containing 5 μL with a concentration of 10^5^ CFU/well was inoculated. After incubation at 37°C for 24 h, 20 μL of a 70% alcoholic solution of INT (0.5 mg/mL) was supplemented in each well and further incubated for 30 min. When the color of the INT changed from yellow to purple in the wells, it meant that microbial growth took place. Three batches were repeated. The MIC values were determined as the minimum concentration of each sample that completely inhibited the growth of microorganisms. The results were expressed in milligrams per milliliter.

### 3.8. Biocompatibility Test

**Hemolysis test** Hemolysis tests were conducted according to previous reports, with some modifications [30]. First, 2 mg a sample was added to PBS to obtain a sample solution with a concentration of 50 μg/mL for 24 h at 37 °C. Then, 0.5 mL of the diluted RBC suspension was mixed with 1.0 mL of the sample solution. PBS and water were set as the negative and positive controls. Following incubation at 37 °C for 1 h, the mixture was centrifuged at 3000 rpm for 5 min, and the absorbance of the supernatant was measured at 540 nm. Each group underwent measurement at least three times. The following formula was used to calculate RBC hemolysis caused by samples:

$$Hemolysis\left(\%\right)=\frac{A_{Sample}-A_{Negative\;control}}{A_{Positive\;control}-A_{Negative\;control}}\times 100\%$$

**Live/dead viability assay of HLF-1 cells** Cell viability was determined using the live/dead cell dye calcein-AM/PI. Briefly, HLF-1 cells were seeded in a dish at a density of 1 × 10^5^ cells per dish and cultured in Dulbecco’s Modified Eagle Medium (DMEM) with 10% fetal bovine serum (Gibco, Carlsbad, CA, USA) and 0.5 mg/mL penicillin-streptomycin for 24 h at 37 °C in 5% CO_2_. The medium was sucked out and replaced with a different sample medium suspension (the concentration of each sample was 0.64 mg/mL). Negative control tests were performed without any samples present. After an additional 24 h, the medium was removed and rinsed from each dish 5 times with a PBS solution before staining the cells with a live/dead cell dye known as calcein-AM/P. Images capturing live cells (green fluorescence) and dead cells (red fluorescence) were acquired using a Zeiss LSM 880 confocal laser (Jena, Germany) scanning microscope equipped with a 10× objective lens.

### 3.9. Statistical Analysis

The mean ± standard deviation (SD) was used to present the experimental results statistically. A one-way analysis of variance (ANOVA) was employed to compare the differences between multiple groups. ** *p* < 0.01 and *** *p* < 0.001 were considered to be statistically significant.

## 4. Conclusions

In conclusion, a series of DB-loaded MSNs (5DB-MSN, 10DB-MSN, and 20DB-MSN) were prepared by simply dispersing DB in MSNs with a pore size of 4 nm and a particle size of 50 nm. DB-MSN could quickly release DB and then activate the intrinsic blood coagulation cascade. Based on the hemostatic advantages of DB and MSN, DB-MSN exhibited remarkably improved properties compared with MSNs and DB, especially 10DB-MSN. In addition, the antibacterial activities and biocompatibility of 10DB−MSN were commendable. The proposed DB-loaded MSN, with rapid hemostasis and antibacterial activities, has potential applications for hemorrhage control.

## Figures and Tables

**Table 1:** Textural properties of MSNs and DB-MSN.

	S_BET_ (m^2^/g)	V_t_ (cm^3^/g)	D_BJH_ (nm)
MSNs	702	2.27	12.96
5DB-MSN	567	2.06	11.43
10DB-MSN	427	1.70	9.47
20DB-MSN	248	0.92	5.43

## Data Availability

All the data are shown in the main manuscript.

## Supplementary Materials

The following supporting information can be downloaded at: https://www.mdpi.com/article/10.3390/molecules29081888/s1, Figure S1: Standard curve of Loureirin A; Figure S2: Standard curve of Loureirin B; Figure S3: (A) UV–vis absorption spectra and (B) corresponding standard curve of DB. Table S1: MIC of different samples.

## References

[1] Spahn D.R., Bouillon B., Cerny V., Duranteau J., Filipescu D., Hunt B.J., Komadina R., Maegele M., Nardi G., Riddez L. (2019). The European guideline on management of major bleeding and coagulopathy following trauma. Crit. Care.

[2] Eastridge B.J., Holcomb J.B., Shackelford S. (2019). Outcomes of traumatic hemorrhagic shock and the epidemiology of preventable death from injury. Transfusion.

[3] Franke A., Bieler D., Friemert B., Schwab R., Kollig E., Gusgen C. (2017). The first aid and hospital treatment of gunshot and blast injuries. Dtsch. Arztebl. Int..

[4] Zhang S.X., Lei X.X., Lv Y.L., Wang L., Wang L.N. (2024). Recent advances of chitosan as a hemostatic material: Hemostatic mechanism, material design and prospective application. Carbohydr. Polym..

[5] Yang G.H., Huang Z.Z., McCarthy A., Huang Y.Y., Pan J.Y., Chen S.X., Wan W.B. (2023). Super-Elastic Carbonized Mushroom Aerogel for Management of Uncontrolled Hemorrhage. Adv. Sci..

[6] Huang X.W., Zheng Y.K., Ming J.F., Bai S.M. (2023). Natural polymer-based bioadhesives as hemostatic platforms for wound healing. Int. J. Biol. Macromol..

[7] Mu K.L., Liu Y.C., Liu G., Ran F., Zhou L.L., Wu Y.T., Peng L.Q., Shao M.H., Li C.J., Zhang Y.P. (2023). A review of hemostatic chemical components and their mechanisms in traditional Chinese medicine and ethnic medicine. J. Ethnopharmacol..

[8] Ohkura N., Yokouchi H., Mimura M., Nakamura R., Atsumi G. (2015). Screening for hemostatic activities of popular Chinese medicinal herbs in vitro. J. Intercult. Ethnopharmacol..

[9] Han B.C., Shen L.D., Xie H.B., Huang Q., Zhao D., Huang X.J., Chen X., Li J. (2023). Synthesis of Carbon Dots with Hemostatic Effects Using Traditional Chinese Medicine as a Biomass Carbon Source. ACS Omega.

[10] Yang B.R., Yuen S.C., Fan G.Y., Cong W.H., Leung S.W., Lee S.M. (2018). Identification of certain Panax species to be potential substitutes for *Panax notoginseng* in hemostatic treatments. Pharmacol. Res..

[11] Liang P., Bi T., Zhou Y.N., Wang C.M., Ma Y.N., Xu H.P., Shen H.P., Ren W., Yang S.J. (2023). Carbonized platycladus orientalis derived carbon dots accelerate hemostasis through activation of platelets and coagulation pathways. Small.

[12] Zhang Q., Qi C., Wang H., Xiao X.F., Zhang Y., Gu S.J., Zhou Y.S., Wang L., Yang H.J., Xu W.L. (2019). Biocompatible and degradable *Bletilla striata* polysaccharide hemostasis sponges constructed from natural medicinal herb *Bletilla striata*. Carbohydr. Polym..

[13] Pona A., Cline A., Kolli S.S., Taylor S.L., Feldman S.R. (2019). Review of future insights of Dragon’s Blood in dermatology. Dermatol. Ther..

[14] Fan J.Y., Yi T., Sze-To C.M., Zhu L., Peng W.L., Zhang Y.Z., Zhao Z.Z., Chen H.B. (2014). A systematic review of the botanical, phytochemical and pharmacological profile of *Dracaena cochinchinensis*, a plant source of the ethnomedicine “dragon’s blood”. Molecules.

[15] Gupta D., Gupta R.K. (2011). Bioprotective properties of Dragon’s blood resin: In vitro evaluation of antioxidant activity and antimicrobial activity. BMC Complement. Altern. Med..

[16] Namjoyan F., Kiashi F., Moosavi Z.B., Saffari F., Makhmalzadeh B.S. (2016). Efficacy of Dragon’s blood cream on wound healing: A randomized, double-blind, placebo-controlled clinical trial. J. Tradit. Complement. Med..

[17] Liu C.Y., Yao W.H., Tian M., Wei J.N., Song Q.L., Qiao W.H. (2018). Mussel-inspired degradable antibacterial polydopamine/silica nanoparticle for rapid hemostasis. Biomaterials.

[18] Kudela D., Smith S.A., May-Masnou A., Braun G.B., Pallaoro A., Nguyen C.K., Chuong T.T., Nownes S., Allen R., Parker N.R. (2015). Clotting activity of polyphosphate-functionalized silica nanoparticles. Angew. Chem. Int. Ed..

[19] Wang C.W., Zhou H.Y., Niu H.Y., Ma X.Y., Yuan Y., Hong H., Liu C.S. (2018). Tannic acid-loaded mesoporous silica for rapid hemostasis and antibacterial activity. Biomater. Sci..

[20] Chen Z.H., Han L., Liu C.J., Du Y., Hu X., Du G., Shan C., Yang K., Wang C.L., Li M.G. (2018). A rapid hemostatic sponge based on large, mesoporous silica nanoparticles and N-alkylated chitosan. Nanoscale.

[21] Huang L.B., Cao K., Hu P., Liu Y. (2019). Orthogonal experimental preparation of Sanguis Draconis-Polyvinylpyrrolidone microfibers by electrospinning. J. Biomater. Sci. Polym. Ed..

[22] Zhang Y.Z., Wang J.C., Bai X.Y., Jiang T.Y., Zhang Q., Wang S. (2012). Mesoporous silica nanoparticles for increasing the oral bioavailability and permeation of poorly water soluble drugs. Mol. Pharm..

[23] Cao L.D., Zhang H.R., Cao C., Zhang J.K., Li F.M., Huang Q.L. (2016). Quaternized chitosan-capped mesoporous silica nanoparticles as nanocarriers for controlled pesticide release. Nanomaterials.

[24] Zhang Y.Z., Zhi Z.Z., Jiang T.Y., Zhang J.H., Wang Z.Y., Wang S.L. (2010). Spherical mesoporous silica nanoparticles for loading and release of the poorly water-soluble drug telmisartan. J. Control. Release.

[25] Yoshida T., Yoshioka Y., Morishita Y., Aoyama M., Tochigi S., Hirai T., Tanaka K., Nagano K., Kamada H., Tsunoda S. (2015). Protein corona changes mediated by surface modification of amorphous silica nanoparticles suppress acute toxicity and activation of intrinsic coagulation cascade in mice. Nanotechnology.

[26] Zheng X.K., Chen L.J., Zeng W.L., Liao W.L., Wang Z.Y., Tian X.B., Fang R.C., Sun Y., Zhou T.L. (2021). Antibacterial and antibiofilm efficacy of chinese dragon’s blood against staphylococcus aureus isolated from infected wounds. Front. Microbiol..

[27] Nandiyanto A.B.D., Kim S.G., Iskandar F., Okuyama K. (2009). Synthesis of spherical mesoporous silica nanoparticles with nanometer-size controllable pores and outer diameters. Microporous Mesoporous Mater..

[28] Jin J., Xu M., Liu Y.X., Ji Z.X., Dai K.L., Zhang L., Wang L., Ye F., Chen G., Lv Z.B. (2020). Alginate-based composite microspheres coated by berberine simultaneously improve hemostatic and antibacterial efficacy. Colloids Surf. B.

[29] Valgas C., Souza S.M.D., Smânia E.F., Smânia A. (2007). Screening methods to determine antibacterial activity of natural products. Braz. J. Microbiol..

[30] Cheng J., Feng S.B., Han S.L., Zhang X.J., Chen Y.D., Zhou X., Wang R.B., Li X.H., Hu H.Y., Zhang J.X. (2016). Facile assembly of cost-effective and locally applicable or injectable nanohemostats for hemorrhage control. ACS Nano.

