# Supplementary material for: Dragon’s Blood-Loaded Mesoporous Silica Nanoparticles for Rapid Hemostasis and Antibacterial Activity
Source: Molecules. 2024 Apr 21;29(8):1888. doi: 10.3390/molecules29081888 (PMC11054711; doi:10.3390/molecules29081888)
Supplement: Supplementary file 1 [file molecules-29-01888-s001.zip › molecules-2937987-supplementary.pdf]

Article

Dragon's Blood-Loaded Mesoporous Silica Nanoparticles for Rapid Hemostasis and Antibacterial Activity

Cuiyun Yin [1,2], Yihang Li [1,2,*], Jing Yu [1,2], Zhaoyou Deng [1,2], Shifang Liu [1,2], Xuanchao Shi [1,2], Deying Tang [1,2], Xi Chen [1,2] and Lixia Zhang [1,2,*]

[1] Yunnan Branch, Institute of Medicinal Plant, Chinese Academy of Medical Sciences, Jinghong 666100, China; cyyin@implad.ac.cn (C.Y.); jyu@implad.ac.cn (J.Y.); zydeng@implad.ac.cn (Z.D.); sfliu@implad.ac.cn (S.L.); xcshi@implad.ac.cn (X.S.); dytang@implad.ac.cn (D.T.); chenxi@implad.ac.cn (X.C.)

[2] Key Laboratory of Sustainable Utilization of Southern Medicine, Jinghong 666100, China

* Correspondence: yhli@implad.ac.cn (Y.L.); lxzhang@implad.ac.cn (L.Z.)

HPLC conditions

Chromatography was performed on a Shimadzu LC-2030C system. Chromatographic separations were carried out on a Thermo BDS Hypersil C_{18} (4.6 mm × 250 mm, 5 μm). The column oven was maintained at 30℃. The mobile phase was composed of acetonitrile (A) and 0.1% aqueous phosphoric acid (B), and the flow rate was set at 1.0 mL/min following the gradient program: 0-20 min, 30-50%A; 20-45 min, 50%A. The detection wavelength was 275 nm and the injection volume was 4 μL.

The standard solutions of Loureirin A and Loureirin B were prepared with concentrations of 1.0 μg/mL, 5.0 μg/mL, 10.0 μg/mL, 15.0 μg/mL, 20.0 μg/mL, 40 μg/mL and 60 μg/mL, respectively. The standard solutions were stored with protection from light. Using above the chromatographic conditions, the standard samples were measured and the standard curve was obtained using the integrated peak area value as the vertical coordinate (Y) and the standard concentration (μg/mL) as the horizontal coordinate (X).

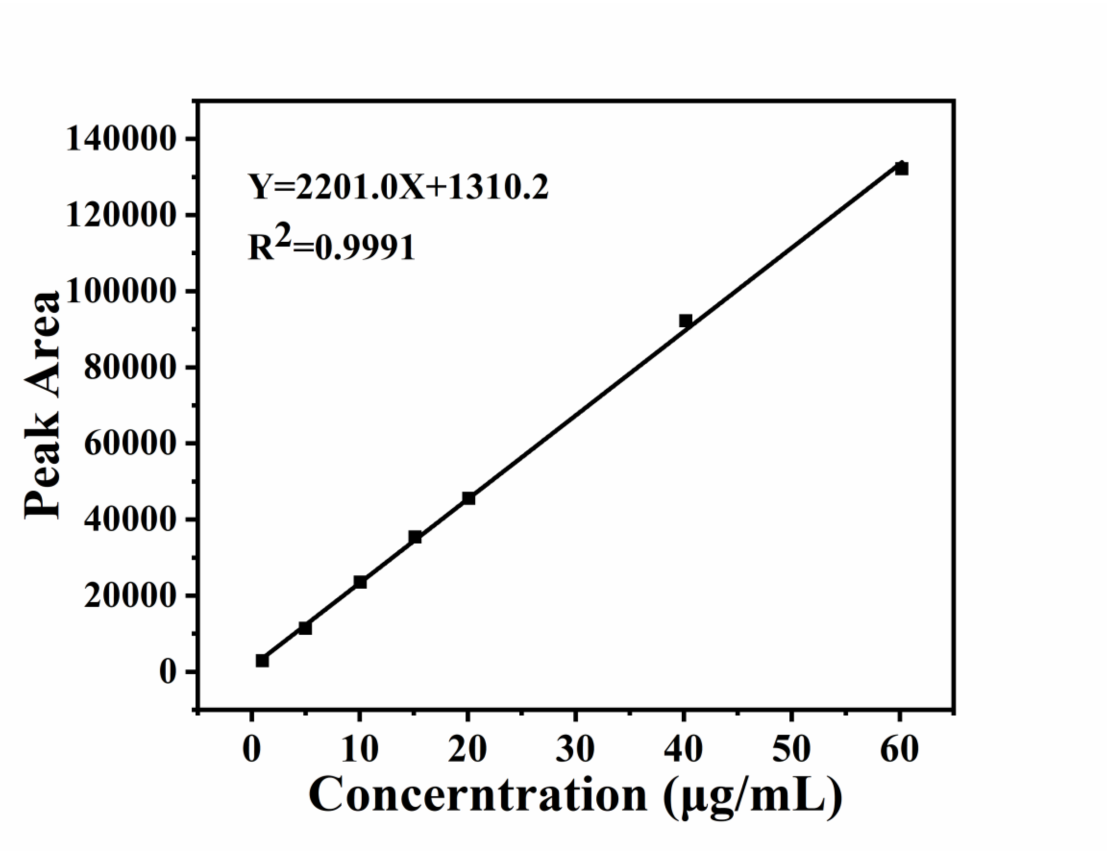

Figure S1 Standard curve of Loureirin A.

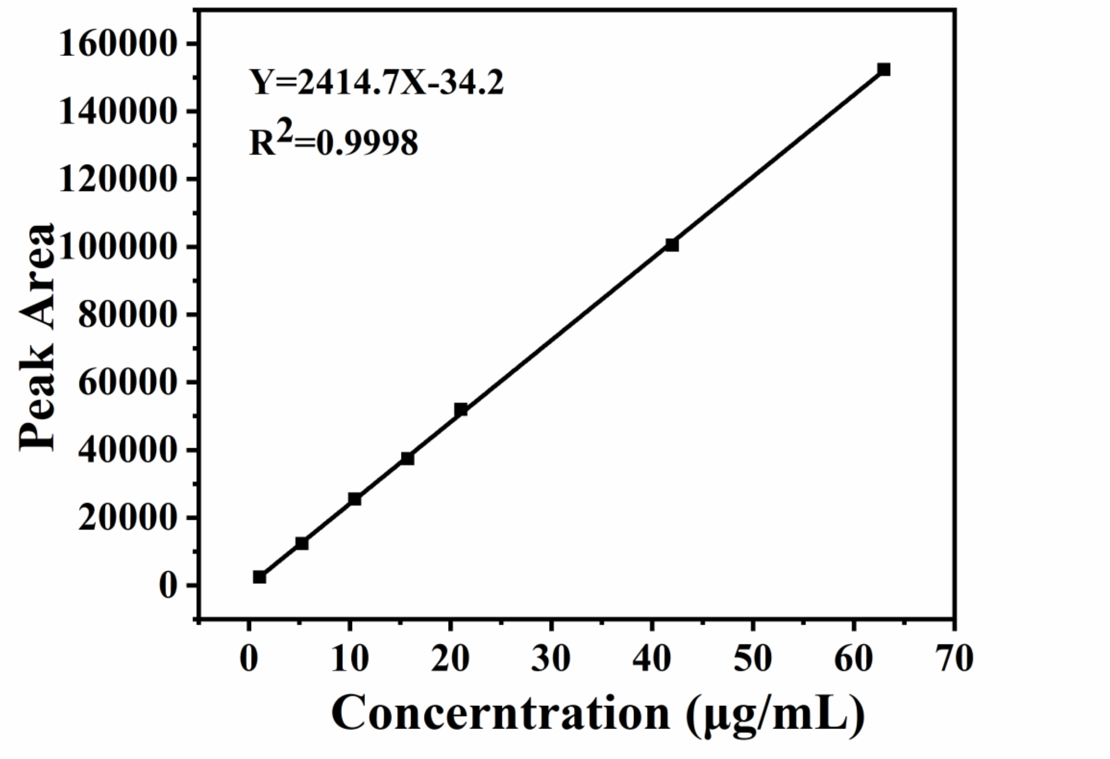

Figure S2 Standard curve of Loureirin B.

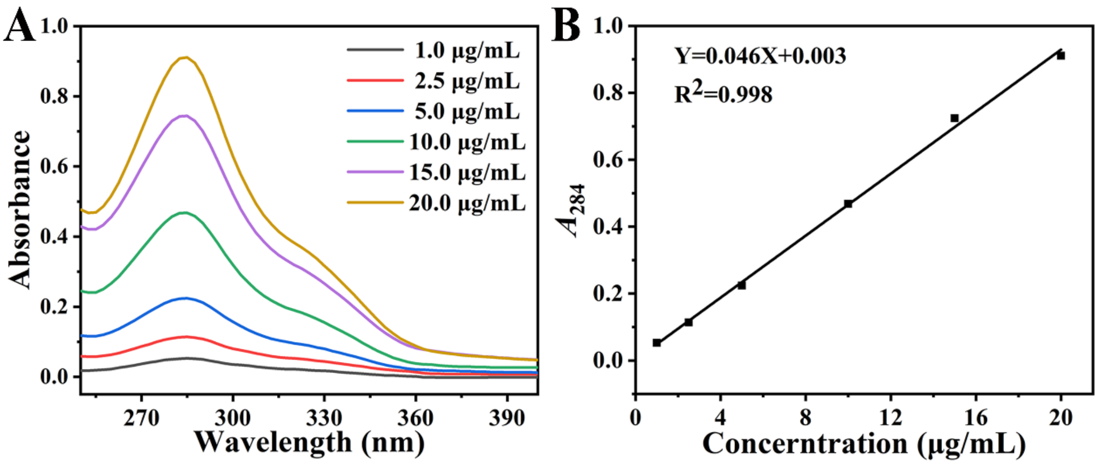

Figure S3 (A) UV-vis absorption spectra and (B) corresponding standard curve of DB.

Table S1 MIC of different samples

samples	MSN	5 DB-MSN	10 DB-MSN	20 DB-MSN	DB
MIC (mg/mL)	>20	10	5	2.5	0.625
